# In Vitro Antimethanogenic Potential of Invasive Weed Species from South-Central Montana in Roughage-Based Diets for Beef Cattle

**DOI:** 10.3390/ani16213466

**Published:** 2026-11-02

**Authors:** Wilmer Cuervo, María Galeano, Casie Flannagan, Elliot Hayhurst, Vidalejandra Araujo

**Affiliations:** 1 Department of Animal and Range Sciences, Montana State University, Bozeman, MT 59717, USA; maria.galeano@montana.edu (M.G.); casie.flannagan@montana.edu (C.F.); vida.araujo@montana.edu (V.A.); 2 Department of Cellular and Molecular Biology, Montana State University, Bozeman, MT 59717, USA; elliott.hayhurst@montana.edu

**Keywords:** digestibility, fermentation, greenhouse gas emissions, plant secondary metabolites, volatile fatty acids

## Abstract

(*1*) *Background:* Beef cattle production is essential for Montana’s economy. These operations rely on fibrous diets promoting enteric methane (CH_4_) emissions, resulting in dietary energy inefficiency and environmental impacts. Concurrently, noxious weeds invade approximately 10% of state rangeland. Yet, some species produce plant secondary metabolites (PSMs) that could be repurposed for CH_4_ mitigation strategies. (*2*) *Methods:* This study evaluated seven invasive weeds from South-Central Montana using in vitro incubations with ruminal fluid from cannulated Angus Cows (590 ± 18 kg). Experiment 1 screened whole plants added at 20% of incubated dry matter (IDM) against a control (hay), then, experiment 2 evaluated ethanolic extracts from leaves and inflorescences from top candidates identified in experiment 1. (*3*) *Results:* In the screening phase, common burdock (BURD) and Curly dock (DOCK) modified fermentation dynamics. BURD leaf extract achieved a 25.2% reduction in CH_4_ yield (mL/g fermented organic matter) without compromising in vitro organic matter digestibility (IVOMD) or ammonia (NH_3_-N). Conversely, DOCK extracts demonstrated the strongest CH_4_ volume reduction but at the cost of a dramatic reduction in IVOMD and NH_3_-N depletion. Bioactivity of PSM remained consistent across anatomical structures. (*4*) *Conclusions*: Specific weeds (BURD, DOCK) represent a biochemical repository with potential as a feed additive for roughage-based diets. Leaves from BURD exhibited optimal potential to mitigate CH_4_ emission without impairing diet digestibility.

## Simple Summary

Beef cattle production is vital to Montana’s Economy, but the ruminal fermentation of cattle produces methane, a potent greenhouse gas that represents a loss of dietary energy. Simultaneously, invasive weeds threaten local pastures, displacing native plants. This study aimed to evaluate seven invasive weeds from South-Central Montana to assess whether the plants or their chemical components could be used as feed additives to reduce methane emissions. Using laboratory simulations of cattle digestion, we discovered that Common burdock and Curly dock altered fermentation, reducing methane production. Likewise, extracts from common burdock achieved a 25% reduction in methane production without affecting the digestion of the diet. Although Curly Dock reduced methane volume, it drastically reduced overall feed digestibility. We concluded that certain invasive weeds can be upcycled as a local feed additive to reduce greenhouse gases from livestock production. These findings may benefit society by offering ranchers a cost-effective strategy to improve cattle efficiency while creating an innovative use of invasive weeds, fostering environmental sustainability.

## 1. Introduction

Among agricultural activities, beef cattle production is a cornerstone of Montana’s economy [1], serving as a vital provider of high-quality protein for humans [2] and economic stability for rural communities in the western United States [3]. Beef cattle operations in Montana heavily rely on extensive systems dominated by native perennial rangeland forage [1]. Despite the economic relevance of this activity, ruminant production faces significant environmental scrutiny due to the generation of enteric methane (CH_4_) emissions [4]. Methanogenesis is a natural byproduct of microbial fermentation in the rumen, representing not only a significant contributor to anthropogenic greenhouse gas emissions but also a major energetic loss for the animal, representing in extreme cases up to 12% of the dietary energy consumed by ruminants [5,6]. Fibrous-based diets typical of semi-arid regions (commonly housing cow-calf operation based on grazing) inherently promote higher acetate to propionate (A:P) ratio in the rumen, shifting metabolic hydrogen pathways toward methanogenic pathways, leading to elevated CH_4_ production per g of organic matter digested [7]. Consequently, finding sustainable, local, and cost-effective nutritional interventions to mitigate enteric emissions in grazing-based operations without harnessing fiber digestibility is an ongoing priority for modern animal science [8].

Concurrent with the CH_4_ mitigation challenge, rangeland degradation driven by the encroachment of noxious weeds poses a massive ecological and economic threat to livestock producers [9]. In Montana alone, approximately 10% of the land area is heavily invaded by noxious and invasive weeds [10], which displace native forage, crops, lower biodiversity, and disrupt natural grazing patterns [11,12]. Interestingly, many of these aggressive plant species possess effective chemical defense mechanisms, characterized by elevated concentrations of bioactive plant secondary metabolites (PSMs), including phenolic compounds, flavonoids, and condensed tannins [13,14]. In ruminant nutrition, isolated PSM from invasive weeds have demonstrated significant antimethanogenic bioactivity [15], either via direct toxicity to the cell membranes of methanogenic archaea [16,17] or by redirecting metabolic hydrogen toward alternative electron sinks like propionate synthesis by ruminal microbes [18,19]. Previous studies in subtropical invasive pigweed (*Amaranthus spinosus*) associated these shifts with an elevated concentration of PSM in aerial components of the plant [15].

Despite its status as a noxious weed (Priority 2B) in Montana, previous studies on Canada thistle (*Cirsium arvense*) report agrochemical and medical potential bioactive PSM, including alkaloids and flavonoids with antimicrobial properties [20,21,22]. Similarly, Common Burdock (*Arctium minus*), categorized as a Priority 3 regulated plant in Montana [23], synthesizes phytochemicals with known antiparasitic and anti-inflammatory capabilities [24]. Curly Dock (*Rumex crispus*) is a non-native perennial spanning a massive breeding range covering nearly 100% of Montana’s suitable habitat [25] and has been widely studied due to the elevated content of Anthraquinones and flavonoids with reported pharmacological applications [26]. Another extremely challenging weed (due to its widespread herbicide resistance) with presence in Montana is Kochia (*Bassia scoparia*), with proven wound-healing and anti-inflammatory effects [27] and is occasionally utilized as “emergency drought forage” in areas with elevated pH in the soil [25]. Equally common, Lambsquarter (*Chenopodium album*) poses a challenge for Montana growers since it behaves as a fierce competitor against pulse and cereal crops [28], yet it contains essential amino acids, key minerals (Fe, Zn), and optimal omega-3 fatty acid levels [29]. Of special interest for Montana, Russian Thistle (*Salsola tragus*) displays mechanisms of resilience against climate change [30], being an introduced species with presence in 54% of suitable habitat statewide [31]. In contrast, previous reports on Prostate Pigweed (*Amaranthus albus*) indicated that young leaves from this species have been used as a direct substitute for spinach, while its seeds can be ground to produce bread-making flour [32] despite containing considerable amounts of phenols and flavonoids in the Amaranthus genus [15].

While commercial synthetic additives and cultivated bioactive plants have been extensively scrutinized for CH_4_ reduction [33], locally and naturally occurring invasive weeds in northwestern temperate grazing systems have received virtually no attention as potential feed additives. Livestock producers frequently manage these invasive species through costly chemical or mechanical eradication [34], overlooking their potential value as thriving species under water stress, reduced nutrient availability [35], and as a freely available chemical “repository” for modulating ruminal fermentation patterns [36]. Furthermore, the variation in concentration and distribution of bioactive PSM on different anatomical portions, as well as the extraction efficiency from these species, remains poorly understood in the context of temperate invasive weeds. Thus, we hypothesized that specific invasive weed species from South-Central Montana with reported content of phenols, tannins, or flavonoids can alter the ruminal fermentation pathways and reduce in vitro CH_4_ yield when added to roughage-based diets. Moreover, we theorize that extracts from different anatomical portions (leaves and inflorescence) will show distinct bioactivity profiles due to differential PSM accumulation. Therefore, the primary objective of this study was to screen seven prominent invasive weed species from south-central Montana and characterize their chemical profiles and impacts on in vitro ruminal fermentation dynamics, volatile fatty acid (VFA) patterns, and methane production and yield. The secondary objective of the current study was to evaluate whether the effects on methanogenesis in the top-performing plant species (in the first experiment) were driven by their specific anatomical portions, when added to the in vitro incubation as ethanolic extracts.

## 2. Materials and Methods

### 2.1. Location and Experimental Animals

This study was conducted at the Bozeman Agricultural Research and Teaching—BART farm, Montana State University, Bozeman, MT, USA. All research procedures were reviewed and approved by the Agricultural Animal Care & Use Committee (protocol number 2025-412-AA). A total of 3 ruminally cannulated commercial Angus cows (590 ± 18 kg of BW; 8 years old) grazing perennial Bluebunch wheatgrass (*Pseudoroegneria spicata*) and meadow hay were included in the study as ruminal fluid donors.

### 2.2. Experimental Design

#### 2.2.1. Experiment 1 Description

Sampling and collection of plant samples were performed at the Oscar Thomas Nutrition Center at BART from a total of 4 mature plants (per species) of weeds of concern with a detected presence in the south-central region of Montana. The selected species were Canada Thistle (*Cirsium arvense*; CAN), Common Burdock (*Arctium minus*; BURD), Curly Dock (*Rumex crispus*; DOCK), Kochia (*Bassia scoparia*; KOC), Lambsquarter (*Chenopodium album*; LAMB), Russian thistle (*Salsola tragus*; RUSS), and Prostate pigweed (*Amaranthus albus*; PIG). A total of four plants were manually harvested in late summer (August) of 2025, and their chemical composition and biomass yield data are shown in Table 1. Samples from each species were dried (65 °C—48 h), ground (2 mm) using a Wiley-type Mill (Arthur H. Thomas Co., Philadelphia, PA, USA), and incubated in a series of five independent in vitro batch culture incubations, performed once monthly from September 2025 to February 2026. After each incubation (24 h), ruminal pH, gas production, ammonia nitrogen (NH_3_-N), methane (CH_4_) concentration, and volatile fatty acids (VFAs) were determined (see Section 2.3 and Section 2.4). Similarly, in vitro organic matter digestibility (IVOMD) was determined (see Section 2.4). Considering that previous studies [15] observed a linear relationship between the addition of the whole plant of invasive weeds and methane mitigation, a 20% inclusion was considered for this exploratory experiment to validate such a linear effect at increasing levels of the whole plant.

#### 2.2.2. Experiment 2 Description

After establishing the methane mitigation potential from the evaluated species in experiment 1, two morphological portions (leaves, inflorescence) from the top two selected species (based on methane mitigation potential; BURD and DOCK) were isolated, dried, ground, and added at 20% of the incubated dry matter (IDM) in a second series of three in vitro batch culture incubations. The same cannulated cows under the same grazing conditions were included as donors of ruminal fluid, which was collected, composited, diluted, and used as the inoculum for the incubations. After each incubation, ruminal parameters were determined in the same way as described in experiment 1. The evaluated morphological portions were selected based on previous studies reporting a greater concentration of polyphenols from invasive species in leaves and seeds or inflorescence [15]. In that study, authors added extracts up to 10% of IDM; this inclusion was utilized because of the elevated recovery yield in the extracts.

### 2.3. Ruminal Fluid Collection and Processing Materials for Batch Culture

The inoculum for all incubations was ruminal fluid (RF) collected from ruminally cannulated cows at 08:00 am in individual pens from the Oscar Thomas Center (BART farm), obtaining ruminal content samples from the medium ventral sac of the rumen as a representative sample of ruminal content. This RF was strained through 4 layers of cheesecloth until RF overflowed a 750 mL pre-warmed (39 °C) thermos to maintain anaerobic conditions. After transporting the samples to the Animal Nutrition Lab, ruminal fluid from the cannulated animals was composited in a 2L Erlenmeyer flask (United Scientific, cat. no. FG4980-250, Libertyville, IL, USA), filtered through 2 layers of cheesecloth, and constantly flushed with CO_2_ to maintain anaerobic conditions in the inoculum. Then, pH was recorded on fresh filtered RF using a manual pH meter (Thermo Scientific VSTAR10 Versa Star, Waltham, MA, USA). Then, RF was diluted (3:1 v:v) with pre-warmed McDougall’s buffer. This inoculum (RF + Buffer) was independently prepared for each incubation. Fifty mL of the inoculum was placed in 125 mL clear, Borosilicate serum bottles (Wheaton, DWK Life Sciences, Vineland, NJ, USA) without diet (blank), containing 0.7 g of dried or containing ground meadow hay stated as control (CON), or 0.56 g of hay plus 0.14 g of the evaluated weed species (20% of IDM). Finally, 125 mL bottles were constantly gassed with CO_2_ before being closed by using metallic crimper seals with rubber septa (Sigma-Aldrich, cat. no. 27454-U, Burlington, MA, USA) to ensure anaerobic conditions during the fermentation process. For experiment 1 (Section 2.2.1), three bottles per species per incubation run were incubated at 39 °C under agitation (60 rpm) for 24 h. Ruminal pH, VFA, NH_3_-N, CH_4_, and gas production were determined after 24 h.

#### Gas Production and Methane Concentration Using a Portable Methane Sensor

Once the incubation was finalized, the fermentative process in the 125 mL glass bottles was stopped by placing them for on an ice bath, gas pressure was determined by using a digital pressure gauge (Grainger International Inc., Lake forest, IL, USA), and a gas sample was collected from the headspace of the bottle using a 14-gauge needle connected to a 2-way valve on a syringe. After removing the needle, the whole gas content was directly inserted from the syringe into the automated collection pump from a portable gas analyzer (Model SKZ1050E-CH4, SKZ Industrial Co., Ltd., Jinan, Shandong, China), and the concentration of CH_4_ (%LEL) was recorded. Following the manufacturer’s instructions, the equipment was calibrated monthly using a certified calibration gas standard tank (CGA320—Airgas, Radnor Township, PA, USA) containing 60% CH_4_ and 40% CO_2_. Calibration standards were prepared from the certified gas tank and included nominal CH_4_ concentrations of 1, 2, 3, 4, 5, 10, 20, 40, and 60%. Each standard was analyzed with the portable sensor, and the observed sensor response was recorded as a percentage of the lower explosive limit (%LEL). The relationship between the known CH_4_ concentration of each calibration standard and the corresponding sensor response was evaluated by linear regression. Calibration performance was assessed using the root mean square error (RMSE), mean bias, relative mean absolute error (RMAE), and precision of the regression.

### 2.4. Laboratory Analysis

#### 2.4.1. VFA Concentration

Following incubation, 125 mL glass bottles were opened, RF was acidified (1:100 of 20% v:v H_2_SO_4_ solution), and stored at −20 °C for VFA determination in liquid–liquid solvent extraction using ethyl acetate [37]. Thawed samples were centrifuged (10,000× *g*, 15 min, 4 °C); 1 mL of the supernatant was mixed with 2 mL of the internal standard of metaphosphoric (25% *w*/*v*): crotonic (2 g/L) acid solution. After being frozen overnight, samples were thawed and centrifuged (10,000× *g*, 15 min, 4 °C), and the supernatant was transferred into 12 × 75 mm borosilicate glass culture tubes (Fisherbrand; Thermo Fisher Scientific Inc., Waltham, MA, USA) and mixed (1:2) with ethyl acetate. After vortexing (2 s), tubes were rested (5 min) to allow the separation of the ethyl acetate, and the obtained top layer fraction was pipetted out into clear glass vials (9 mm; Fisherbrand; Thermo Fisher Scientific Inc., Waltham, MA, USA). Samples of VFAs were analyzed using a gas chromatography–mass spectrometry system (GC-MS 5977C Series MSD; Agilent Technologies Santa Clara, CA, USA) by separating them on a capillary column (CP-WAX 58 FFAP 25 m × 0.53 mm, Varian CP7767; Varian Inc. Walnut Creek, CA, USA). Helium (He) served as the carrier gas at a constant flow rate of 1.0 mL/min. The injector temperature was set at 200 °C. The column oven temperature was initially held at 110 °C. The MS interface transfer line was kept at 240 °C, while the EI ion source and quadrupole temperatures were set to 230 °C and 150 °C, respectively. To protect the filament from the extraction solvent, a solvent delay of 3 min was applied. Data acquisition was conducted in scan mode across a mass range of m/z 40 to 200 for peak identification and target ion integration.

#### 2.4.2. Ammonia Concentration

Ammonia-N was determined through the phenol-hypochlorite reaction, according to Broderick and Kang [38]. Briefly, RF samples stored at −20 °C were thawed at room temperature and centrifuged (10,000× *g* for 15 min at 4 °C). Then, 20 μL of supernatant was pipetted into 12 × 75 mm borosilicate glass culture tubes (Fisherbrand; Thermo Fisher Scientific Inc., Waltham, MA, USA) containing 1 mL of Phenol reagent. Tubes were vortexed, 0.8 mL of a hypochlorite solution was added, vortexed again, covered with glass marbles, and placed in a water bath at 95 °C for 5 min. Finally, 200 μL from each sample were pipetted in a 96-well plate reader spectrophotometer (Agilent BioTek, Winooski, VT, USA) and the absorbance was recorded at 665 nm.

#### 2.4.3. In Vitro OM Digestibility

Simultaneous with each batch culture incubation, 0.7 g of the substrate (diet plus all the treatments) and inoculum (diluted RF) were incubated in plastic scintillation tubes of 200 mL volume with rubber stoppers to analyze IVOMD following a modified Tilley and Terry procedure [39]. Briefly, After 24 h of incubation, 6 mL of 20% (V:V) HCl solution was added to each tube, and after a slight swirl, 2 mL of a 5% (v:v) pepsin suspension was added, then tubes were returned to the incubator for another 24 h. After this period, samples were deposited in filter paper (Whatman No. 541 ashless; Whatman International Ltd., Maidstone, UK), and filtered, rinsed with deionized water, dried (65 °C for 48 h), and then burned (550 °C, 6 h) to determine IVOMD by gravimetric calculations as follows.

IVOMD (%) = [(incubated organic matter − residual organic matter)/incubated organic matter] × 100.

#### 2.4.4. Extraction from the Vegetative Material

Sub-samples from the dried and ground vegetative material were exposed to a solid–liquid extraction method using a 70% ethanol solution. Briefly, 10 g of dried material from each species were mixed with 250 mL of the extraction solution and placed in an orbital shaker (0180-PRO USALAB, Livonia, MI, USA) at 60 °C, 80 rpm for 2 h. The content was filtered (Whatman No. 3), and the filtrated liquid was stored in 50 mL Falcon tubes for further lyophilization. The solid extracts were stored in Amber Boston Round Glass Bottles at −20 °C [40].

#### 2.4.5. Total Phenols Content and Flavonoid Determination

Total phenolic content in the extracts from the invasive weeds was determined using the Folin–Ciocalteu method as adapted by [41]. The absorbance of each sample was measured in a spectrophotometer at 760 nm (Agilent BioTek, Winooski, VT, USA). Gallic acid (Spectrum Chemical Cat No. G1034, New Brunswick, NJ, USA) was used to generate the standard curve (0, 25, 50, 100, 150, and 200 mg/L), and the total phenolic content was expressed as mg GAE/g DM extract. Total flavonoids were determined according to the AlCl_3_ method [42] by recording absorbance at 367 nm. The calibration curve was prepared with six points (0, 25, 50, 100, 200, and 400 mg/L) using quercetin (QE, Thermo Fisher Scientific, Waltham, MA, USA) and the observed values were expressed in mg QE/g DM extract.

#### 2.4.6. Condensed Tannins Content Quantification

Condensed tannins concentration was estimated using the vanillin-HCl colorimetric assay [43]. Briefly, 0.5 mL of the whole plant extract was mixed with 3 mL of a 4% (*w*/*v*) vanillin (Sigma-Aldrich, Cat. No. V1104) in methanol, followed by a slow addition of 1.5 mL of a concentrated solution (38%) of HCl. After 15 min of incubation at room temperature in an Ambar glass flask, 200 μL per sample were pipetted into a 96-well plate, and absorbance was measured at 500 nm using a UV–Visible spectrophotometer (Agilent BioTek, Winooski, VT, USA). Three additional amber flasks in which vanillin was replaced by methanol were used as blanks to correct for extract color. Catechin standards (Sigma-Aldrich, Cat. No. C125-10G) of 0, 10, 20, 50, 100, and 200 mg/L were prepared in a similar fashion to the samples to generate a calibration curve. The content of condensed tannin content was calculated from the curve and expressed as mg catechin equivalents (CEs) per g of dry plant material, accounting for the extracted volume and dilution.

### 2.5. Calculations and Statistical Analysis

For the CH_4_ calculation, the pressure in each bottle was recorded through a digital gauge, and the recorded pressure (psi) was converted to absolute pressure

$$P_{\mathrm{abs}}=\frac{P_{g}+P_{\mathrm{atm}}}{14.696}$$

where P_abs_ is the absolute pressure (atm), P_g_ is the pressure measured with the gauge in psi, P_atm_ is the atmospheric pressure of the lab (12.3 psi—Bozeman, MT, USA), using 1 atm = 14.696 psi for pressure conversion. For each bottle, total moles of gas in the headspace were then estimated using the ideal gas law, with headspace volume and incubation temperature used as fixed parameters.

*PV* = *nRT*

$$n=\frac{P\times V}{R\times T}$$

where

*n* = Total gas moles;

*P* = P_absolute_;

*V* = Headspace = 0.075 L;

*R* = Gas constant (0.08206 L × atm/mol × K°);

*T* = Temperature (39 °C = 312.15 K°).

After performing the calibration curve of the sensor, the regression values were obtained, including intercept (7.077), slope (10.094), R^2^ (0.998), mean square error (RMSE = 3.611), mean bias (0.3913), and precision (3.10%). Methane readings from the sensor (% LEL) were converted to CH_4_ concentration (% *v*/*v*) in the headspace.

CH4% = (CH4%LEL − 7.077)/10.094

Then, CH_4_ moles were calculated by multiplying total gas moles (*n*) by CH_4_ concentration (% *v*/*v*). Methane production was subsequently expressed as volume (mL) using the molar gas volume calculated at the incubation temperature (39 °C) and measured absolute pressure.

Results from the first experiment were analyzed as a randomized complete block design, using the MIXED procedure of SAS version 9.4 (SAS Institute Inc., Cary, NC, USA). The statistical model included plant species as a fixed effect and incubation run as a random blocking effect. Each incubation represented a block, and the average of the three bottles receiving the same plant species within each incubation was considered the experimental unit. Thus, five independent experimental units for each species and compared using Tukey’s adjustment for multiple comparisons. The first experiment followed this model:

*Y_ij_* = *μ* + *Species_i_* + *Run_j_* + *e_ij_*

where:

*Species* = Fixed;

*Run* = Random block;

*e* = Experimental error.

For Exp 2, the data were analyzed as a randomized complete block design with a 2 × 2 factorial arrangement of treatments consisting of two species, two morphological portions, and species × portion interaction as a fixed effect, with incubation run included as a random blocking effect. The average of the three bottles within each incubation was considered the experimental unit, and data were analyzed according to the following model:

*Y_ijk_* = *μ* + *Species_i_* + *Portion_j_* + (*Species* × *Portion*)*_ij_*+ *Run_k_* + *e_ijk_*

where:

*Species*, *Portion*, *Species* × *Portion* = Fixed effects;

*Run* = Random block;

*e* = Experimental error.

The four extracts were analyzed relative to the control treatment (Hay) using pre-planned comparisons to evaluate the effect of each plant extract treatment relative to the control. Multiplicity was accounted for using the Dunnett adjustment for comparisons of multiple treatments with a common control. The exact treatment comparisons and adjusted *p*-values were obtained using the LSMEANS statement with the ADJUST = DUNNETT order. For both experiments, statistical significance was declared at *p* < 0.05, and tendencies were considered when 0.05 < *p* < 0.1. All response variables, including ruminal pH, gas, CH_4_ concentration, NH_3_-N, VFA, and IVOMD, were analyzed independently using the corresponding model described above. Model assumptions were evaluated by examining the residuals for normality and homogeneity of variance.

## 3. Results

### 3.1. Experiment 1 Results

#### 3.1.1. Secondary Metabolite Characterization

The secondary metabolite profiling revealed notable differences among the evaluated weed species (Table 2). Total phenolic content (TPC) varied significantly across treatments (*p* < 0.001), with BURD exhibiting the highest concentration (360.08 mg GAE/g) followed by DOCK (184.68 mg GAE/g), while CAN exhibited the lowest concentration (28.7 mg GAE/g). Regarding the flavonoid content (TFC) DOCK exhibited the highest concentration (261 mg QE/g), while these compounds were not detected in KOC through the proposed method (AlCl_3_ method). Tannins content (QTV) was strongly affected by species (*p* < 0.001) where DOCK reached peak concentrations, followed by LAMB and BURD (274.39, 222.32, and 180.45 mg CE/g, respectively).

#### 3.1.2. Volatile Fatty Acids Profile

The addition of invasive weeds at 20% of the incubated dry matter (IDM) altered ruminal end-products after 24 h (Table 3). Total VFA concentration decreased after KOC addition (53.88 mM) compared to the control hay (CON; 65.76 mM; *p* = 0.041). Molar proportion of acetate tended to change across species (*p* = 0.069). When compared to CON, KOC addition increased propionate proportion, and PIG decreased it (*p* = 0.049). Consequently, A:P was reduced in bottles receiving KOC (1.7), BURD (2.2), and DOCK (2.1) compared to CON (2.4) and PIG (2.6) (*p* = 0.039). Butyrate and BCVFA molar proportions were highest for KOC and lowest for DOCK, LAMB, RUSS, and PIG (*p* < 0.05).

#### 3.1.3. In Vitro Ruminal Fermentation Parameters

Gas kinetics, NH_3_ concentration, and IVOMD are summarized in Table 4. Neither ruminal pH nor IVOMD were affected by the inclusion of any of the evaluated species during the first screening phase. Likewise, despite numerical differences in BURD and RUSS for IVOMD (48.96% and 47.45%, respectively) compared to 41.31% in CON, no statistical differences were detected (*p* = 0.781). Gas pressure (PSI, *p* = 0.001) and total gas production (*p* < 0.01) were minimized by DOCK addition (11.96 PSI and 4.97 mmol, respectively). Simultaneously, methane volume (mL) dropped notably (*p* < 0.001) after DOCK, BURD, LAMB, and RUSS addition (0.72, 0.81, 0.81, and 0.80 mL, respectively) compared to CON (0.87 mL). While CH4 normalized by IDM (mL/f IDM) showed no differences among species (*p* = 0.398), CH_4_ yield (mL/g OMF) was reduced (*p* < 0.01) in 21.5 and 20% by BURD, RUSS compared to CON, closely followed by DOCK (2.47 mL/g OMF) while KOC seemed to increase methanogenesis (3.06 mL/g OMF). Based on their combined capacity to mitigate CH_4_ yield without restricting OM digestibility, greater propionate concentration, and promoting a lower A:P ratio, BURD and DOCK were selected as the top candidate species for further morphological evaluation in experiment 2.

### 3.2. Experiment 2 Results

The central aim of this screening was to evaluate whether the anti-methanogenic bioactivity of BURD and DOCK (selected in experiment 1) is influenced by the specific morphological portion (leaves vs. inflorescence) when supplied as ethanolic extracts. The extraction yield showed a statistical tendency to differ among species and portions (Table 5; *p* = 0.058), with BURD-leaf and DOCK-inflorescence extracts showing higher yields (107.31 and 111.32 mg/g, respectively).

Phenolic and tannin concentrations were influenced by species and portion, whereas total flavonoids and extract yield were comparatively stable. A Species × portion interaction was detected on the total phenols (TPC; *p* < 0.01), with the greatest concentration observed in BURD leaves (158.83 mg GAE/g) and the lowest concentrations in DOCK leaves and inflorescences (47.88 and 56.18 mg GAE/g, respectively). Phenols (TPC) decreased from leaves to inflorescences extract in BURD but slightly increased in DOCK. Likewise, tannins (QTV) concentration was affected by a Species × portion interaction (*p* < 0.01), with the extracts from DOCK inflorescences showing the greatest concentration (157.08 mg CE/g). Both Yield (mg/g) and flavonoid content (TFC) were not affected by species, portion, or their interaction (*p* > 0.05). Overall, the significant species-portion interactions for TPC and QTV may suggest that the distribution of these secondary metabolites between leaves and inflorescences substantially differs between BURD and DOCK.

#### 3.2.1. VFA Concentration After the Addition of Extracts

As indicated in Table 6, there was a significant species × portion interaction for propionate concentration (*p* = 0.008) and total VFA (*p* = 0.001). Pre-planned contrasts confirmed that total VFA and propionate concentration increased dramatically (*p* < 0.01) for all the evaluated extracts compared to CON, with BURD-leaf extract addition leading to the highest propionate value (19.45 mM). Branched chain VFA (BCVFA) was reduced after the inclusion of both extracts (leaf, infl) from DOCK (*p* = 0.02). Regarding molar proportions, a notable main factor effect of the portion (*p* < 0.01) was observed on all evaluated VFA. While the addition of leaf extracts from both species mediated an increase in propionate and Butyrate (*p* < 0.01), the inclusion of inflorescence promoted a reduction in BCVFA. Thus, the addition of leaf extracts seemed to shift fermentation pathways toward a more efficient profile by decreasing acetate from 62.12 mol/100 moles (CON) to less than 54 mol/100 moles and boosting propionate proportion from 24.25 (CON) up to 32.7 mol/100 moles.

#### 3.2.2. Fermentation Patterns and Methane Mitigation Under Extracts Inclusion

As compiled in Table 7, except for methane yield, no further significant species × portion interactions were detected for the rest of the ruminal fermentation parameters (*p* > 0.49). Nevertheless, pre-planned contrasts verified that every single extract treatment decreased gas pressure, total gas, and CH_4_ volume (*p* < 0.01) compared to CON. The notable species effect revealed that both extracts from DOCK exerted a stronger mitigation effect on gas pressure (40%), total gas (21%), CH4 volume (25%), and production (25%) compared to CON. Mitigation from BURD extracts on the same parameters was below 20%, compared to CON values.

A significant species × portion interaction was detected for CH_4_ yield (mL/g FOM; *p* = 0.021). All evaluated extracts reduced this parameter relative to CON (*p* < 0.01), yet BURD leaf extract triggered the lowest methane yield (2.58 mL/g FOM), representing a 25.2% reduction relative to CON. The main effect of portion affected pH values (*p* < 0.01), with inflorescence extracts leading to a significant increase in pH after those extracts were added to the ruminal fluid (*p* < 0.01). When compared to CON, NH_3_-N concentration was notably diminished by either of the DOCK extracts (*p* = 0.046). Similarly, IVOMD was markedly decreased when both extracts from DOCK were added to the substrate, while BURD extracts preserved or numerically enhanced digestibility (up to 45.13%).

## 4. Discussion

The search for local, cost-effective, and natural feed additives that mitigate enteric CH_4_ emissions without affecting the digestibility of a diet represents a cornerstone of sustainable beef cattle systems [7]. In South-Central Montana, heavy encroachment of crops and prairies with invasive weeds simultaneously represents an ecological challenge [9] and an unexplored chemical repository. The screening approach proposed in the first experiment and the targeted morphological extract evaluations in the second experiment allowed us to test the hypothesis about the role of secondary metabolites from BURD and DOCK in changes in ruminal fermentation dynamics and methanogenesis.

### 4.1. Phytochemical Profiles and In Vitro Fermentation Pathways

The secondary metabolites (Flavonoids, tannins, total phenols) detected in this study align with the botanical defense mechanisms previously reported in similar species [13,44]. Thus, DOCK exhibited exceptionally higher flavonoid concentration (>260 mg QE/g) and condensed tannins (>270 mg CE/g), in agreement with previous studies [45,46]. Tannins have been widely recognized in ruminant nutrition for their ability to form reversible complexes with macronutrients and microbial cell walls [47]. In Exp. 1, DOCK inclusion lowered total gas production and numerical NH_3_ concentration, which corresponded to DOCK extracts reducing NH_3_ concentration to 4.6 mM in Exp. 2. This redundant outcome has been previously identified as a “protein-sparing” effect after tannins are introduced into the ruminal environment, which prevents excessive proteolysis and subsequent deamination by hyper-ammonia-producing bacteria [48].

A critical trade-off was identified in Exp. 2 when DOCK extracts caused a sharp decrease in IVOMD (to 35%). This negative response matches previous in vitro findings where elevated concentrations of condensed tannins in phenolic fractions directly impaired the attachment activity of cellulolytic bacteria such as *Fibrobacter succinogenes* and *Ruminococcus albus* [49,50]. Similarly, this outcome has been associated with the chemical binding of tannins to structural carbohydrates of the forage, reducing overall dry matter disappearance [47]. This coincided with the elevated condensed tannins concentration detected in the DOCK-inflorescence extract (157.08 mg CE/g).

Conversely, BURD (*Arctium minus*) displayed a unique behavior by exhibiting the highest phenols content (360.08 mg GAE/g) but lower tannin thresholds compared to DOCK. Interestingly, BURD inclusion preserved or increased IVOMD in both experiments, reaching up to 48.96% in the screening phase (Exp. 1). A previous report indicated that phenols in DOCK exhibited an elevated content of lignans, arctiin, and hydroxycinnamic acids such as chlorogenic acid [51]. This precedent, along with our observations, suggests that the specific phenolic architecture of DOCK may not represent a toxic effect on core fibrolytic populations. Instead, it may function similarly to certain essential oils or non-tannin phenolics that selectively impact inefficient microbial groups [52] while leaving primary organic matter degraders almost intact. Further microbiome dynamics analyses are required to address this hypothetical mechanism.

### 4.2. Methane Mitigation Mechanisms

Enteric CH4 mitigation via secondary metabolites is typically mediated through two modes of action: direct toxicity to the membrane of methanogenic archaea [33] or indirect inhibition via redirection of metabolic hydrogen (H2) away from the methanogenic pathway and towards sinks, such as propionic–genic pathways [53]. The VFA profiles observed in this study seemed to support the indirect pathway framework. In both experiments, the introduction of the selected invasive species or their ethanolic extracts caused a dramatic shift in VFA dynamics, characterized by a sharp drop in A:P ratio. This shift was evident in Exp. 2, where propionate proportion went from 24.25 mol/100 mol in CON up to 32 mol/100 mol under leaf extracts from both species. Since the biochemical synthesis of propionate consumes metabolic hydrogen [54], it directly competes with methanogenesis for the available H_2_ in the ruminal pool [55].

When calculating mitigation efficiency per unit of fermented organic matter (mL of CH4/ g FOM), BURD and DOCK proved to be the most promising candidates among the selected species for Exp. 1. In Exp. 2. BURD leaf extract produced the lowest methane yield (25.2% reduction) among the evaluated extract treatments while maintaining IVOMD relative to the control. The observed results in Experiment 2, showing the indistinct restriction of the absolute CH_4_ across the evaluated extracts from leaves and inflorescence tissues, suggest that bioactive agents are uniformly distributed across the upper anatomical structures in these mature plants harvested in the late summer of Montana. This fact represents a practical advantage for future harvesting or grazing management strategies, as whole-top mechanical collection would be viable. While the utilized in vitro systems offer an essential high-throughput tool to isolate bioactive candidates [56], they lack the absorptive capacity, continuous passage rates, and long-term microbial adaptation characteristic of a live animal [57]. The depression of digestibility with DOCK highlights the relevance for cautious extraction and dosing strategies; optimizing inclusion levels below 20% of IDM might help retain the anti-methanogenic potential while reducing the adverse effects on fiber degradation.

Furthermore, while KOC has been explored as an alternative forage during drought due to its marginal protein content [58], data from the current study indicate it increased CH_4_ yield per g of FOM (3.43) and disrupted overall VFA output (53.88 mM), marking it as an inefficient option for greenhouse gas mitigation strategies. This severe disruption of energetic output can be hypothetically associated with the phytochemical architecture of KOC, which can accumulate exceptionally high concentrations of soluble oxalates, nitrates, and terpenoids in mature plants [44,58]. In this sense, when an unadapted ruminal microbial ecosystem is abruptly exposed to high oxalate concentrations, metabolic pathways might be impacted, potentially leading to toxicity processes affecting fibrolytic microorganisms and shifting fermentative pathway dynamics towards inefficient pathways [9]. Furthermore, advancing maturity dates in KOC are known to cause sharp declines in overall fiber digestibility, imposing a higher accumulation of indigestible structural carbohydrates that slow down passage rates and prolong the time available for methanogens to harvest metabolic hydrogen [55]. Consequently, while KOC remains a resilient biomass source for rangeland survival, the results from the current study explicitly rule it out as a viable CH_4_ mitigation strategy in the western United States. Nonetheless, processing methods for this and the rest of the evaluated invasive species should be considered in future studies as an alternative to modify the secondary metabolites’ architecture to reduce the negative outcomes observed in the current study. Likewise, future studies should prioritize isolating the specific active molecules within BURD leaves and performing continuous culture or in vivo trials to confirm long-term microbial adaptation and palatability. Safety concerns about including the evaluated amount of harvested invasive plants or their extracts should be specifically evaluated using ex vivo approaches, checking changes in ruminal microbiome and animal’s blood parameters and metabolome analyses.

### 4.3. The Critical Balance of Ruminal Ammonia (NH_3_-N)

The addition of BURD leaf extract triggered the most efficient outcome in terms of methane mitigation without affecting IVOMD and maintained NH_3_ levels within the physiological requirements for microbial crude protein synthesis [59]. In contrast, addition of DOCK extracts promoted a dangerous reduction in NH_3_ close to this limit (5 mg/dL~3.57 mM). The dramatic NH_3_ concentration decreases observed when DOCK extracts were added to the bottles might highlight a physiological conflict between methane mitigation and the overall ruminal function. The widely accepted NH_3_ threshold has been identified as the minimum concentration required to prevent restriction on microbial growth and ensure baseline microbial protein synthesis [59,60]. The drop in NH_3_ down to 4.6 mM sits above the absolute biological floor for microbial survival; they almost reach critical levels, which has been associated with limitations in growth of highly inefficient hyper-ammonia-producing bacteria, as well as essential cellulolytic populations [48,50]. This dramatic reduction has been closely related to the elevated concentration of condensed tannins in DOCK inflorescence (157.08 mg CE/g). Previously, elevated contents of condensed tannins have been correlated with a reduced availability of dietary protein in the rumen [48] and thereby lowering NH_3_ concentration, potentially due to physical connection between proteins and tannins [47], and/or membrane damage to ruminal microorganisms in charge of proteolysis [50]. Collectively, the combined observations from methane mitigation and NH_3_ suggest that while DOCK produced the greatest reduction in CH_4_ volume among the evaluated extracts, this response was accompanied by reduced IVOMD and NH_3_-N concentration. Thus, direct supplementation as an additive in low-quality roughage-based diets (i.e., grazing Cow-calf operations in Montana) could potentially trigger severe systemic protein deficiencies unless accompanied by non-protein nitrogen supplementation. This hypothesis must be further addressed through in vivo feeding trials including microbiome analyses to confirm the impact on ruminal populations.

### 4.4. Inclusion Level and Dosage Comparison

When interpreting the antimethanogenic efficacy, it is critical to contextualize inclusion levels under experimental conditions (i.e., 20% of IDM) relative to standard doses of established commercial and synthetic feed additives. Commercial methane inhibitors, such as the synthetic 3-nitrooxypropanol (3-NOP) or concentrated commercial plant extracts from *Yucca schidigera* and *Quillaja Saponaria*, typically exert potent ruminal modifications at significantly lower inclusion thresholds, ranging from less than 0.1% to 5% of the dietary DM [7,33]. Standard applications of industrially purified *Yucca schidigera* extracts deliver specific saponins or non-tannin phenols that depress methanogenesis without dramatically hindering fiber fermentation [55]. In contrast, using an unrefined whole-plant biomass inclusion of 20% of IDM (screening phase in Exp 1) may introduce a bulky matrix of cell-wall fractions along with the target bioactive secondary metabolites. Thus, while crude extract supplementation at that level may pose a risk of decreasing total-tract nutrient digestibility and DM intake in live animals [46,57], it serves as a valid high-throughput diagnostic threshold to isolate high-potency raw biomass candidates. For future practical rangeland management or mechanical harvesting strategies, this 20% threshold must be reassessed and optimized downward (dose–response approach) to pinpoint a localized “sweet spot” that maintains the mitigation efficacy observed here, while avoiding harmful effects on digestibility of nutrients.

### 4.5. Limitations and Future Research

Several limitations of the present study should be considered when interpreting the results. First, both experiments were conducted using ruminal fluid from donor cannulated grazing cows, and such fluid was pooled prior to incubation. Although pooling provided a consistent ruminal environment among bottles within an experimental run, it limited the ability to evaluate donor-to-donor variation and may limit the biological representativeness of the inoculum. Thus, further ex vivo and in vivo studies should be performed to validate the observed outcomes. Fermentation times for batch culture (24 h) and IVOMD procedures (48 h) utilized in the current study have been previously cited by researchers in the field of ruminants in vitro studies [57]. Second, despite being a standardized and accepted protocol, the in vitro batch culture utilized a short incubation period (24 h) and a single forage substrate. Therefore, the observed effects may not fully represent the expected responses under more complex and dynamic conditions of the rumen in vivo. Third, the crude ethanolic extracts were evaluated at a relatively high inclusion (20% of IDM). This rate was selected based on previous similar studies [14] as a screening level to identify potential bioactive effects on ruminal fermentation and CH_4_ production, rather than as a directly applicable commercial supplementation rate. Consequently, the magnitude of the responses should not be interpreted as evidence that 20% inclusion would be practical or appropriate for grazing cattle diets. Dose–response using substantially lower inclusion rates and standardized extract concentrations is needed to identify effective and practically relevant doses. In addition, the present study did not evaluate animal performance, feed intake, performance, toxicity, or potential effects on animal health. The crude extracts were also not chemically standardized for individual bioactive compounds; therefore, the specific compounds or combinations of compounds responsible for the observed responses could not be identified. Furthermore, hydrogen production was not measured, nor were the changes in the abundance or activity of methanogenic archaea characterized. Finally, CH_4_ production was evaluated only under in vivo conditions, and the potential effects of the extracts on enteric CH_4_ emissions under practical conditions remain to be determined. Future research should therefore include dose–response experiments with standardized extracts, characterization and quantification of individual bioactive compounds, assessment of potential mechanisms involving hydrogen and methanogens, and subsequent in vivo studies evaluating CH_4_ emissions, ruminal fermentation, feed intake, animal performance, palatability, and safety.

## 5. Conclusions

This study demonstrated that the selected invasive species with presence in South-Central Montana hold significant, untapped biochemical potential to modulate ruminal fermentation and mitigate CH_4_ emissions. Among the evaluated invasive weeds, BURD and DOCK demonstrated to contain extractable compounds capable of substantially modifying ruminal fermentation and CH_4_ production, and this mitigation response appears to coincide with changes in VFA partitioning (increased propionate, reduced A:P) rather than generalized inhibition of fermentation. Therefore, future studies could focus on establishing whether the underlying mechanism for CH_4_ mitigation mediated by these extracts is driven by a metabolic shift toward propionate synthesis rather than a generalized toxic depression of fermentation by using H_2_ flux measurement, methanogen abundance, and even isotope tracing. Within the species with greater potential, BURD demonstrated potential for future evaluations as a feed additive for extensive beef systems. Extracts from BURD leaves achieved a 25,2% reduction in CH_4_ per unit of fermented organic matter while preserving IVOMD and NH_3_ levels within safe physiological thresholds. Conversely, DOCK exerted the strongest absolute reduction in CH_4_ volume, likely due to its elevated condensed tannins levels triggering a severe trade-off by dramatically reducing IVOMD and depleting NH_3_ levels. Anatomical profiling revealed that the presence of phenols, flavonoids, and condensed tannins is consistent between the whole plant, leaves, and inflorescences in BURD and DOCK mature plants. This suggests that simplified harvesting of aerial portions is viable. Collectively, findings from this study provide initial information about considering noxious weeds as strategic biomass to foster sustainable grazing in cattle operations in the western United States. Yet, future in vivo research is mandatory to validate inclusion levels in feeding trials and to assess processing methods of plant biomass, palatability, and long-term microbial adaptation.

## Figures and Tables

**Table 1:** Chemical composition and biomass yield of invasive weeds in South-Central Montana.

		Invasive Weed Species ^1^						
Item ^2^	HAY	CAN	BURD	KOC	DOCK	PIG	LAMB	RUSS
DM, g/100 g	895.17 ± 23.43	316.67 ± 49.33	230 ± 20	563.33 ± 11.55	256.67 ± 40.41	306.67 ± 35.12	286.67 ± 30.55	396.67 ± 58.59
OM, % of DM	89.01 ± 1.47	89.51 ± 5.61	89.53 ± 7.20	89.00 ± 7.21	90.47 ± 8.97	89.67 ± 8.99	88.31 ± 7.45	88.48 ± 7.83
CP, % of DM	9.01 ± 0.92	19.99 ± 1.52	13.0 ± 2.6 d	15.0 ± 1.73	18.0 ± 1.01	19.33 ± 1.52	19.9 ± 1.01	11.33 ± 0.57
NDF, % of DM	52.61 ± 11.04	46.0 ± 26.46	52.33 ± 5.77	55.33 ± 2.51	46.67 ± 2.87	44.67 ± 5.77	45.33 ± 2.08	52.33 ± 2.31
ADF, % of DM	32.38 ± 3.78	37.3.33 ± 2.08	42.01 ± 2.64	41.01 ± 2.64	34.67 ± 1.15	35.33 ± 3.05	35.10 ± 1.73	44.67 ± 3.51
Biomass, g DM/plant	-	250 ± 41	510 ± 62	198 ± 24	150 ± 19	59 ± 19	210 ± 25	650 ± 89
Density, plants/m^2^	-	3	4	5	8	6	5	1

^1^ HAY = Meadow grass hay. CAN = Canada Thistle. BURD = Common Burdock. KOC = Kochia. DOCK = Curly Dock. PIG = Prostate pigweed. LAMB = Lambsquarter. RUSS = Russian thistle. ^2^ DM = Dry matter. OM = Organic Matter. CP = Crude protein. NDF = Neutral Detergent Fiber. ADF = Acid detergent fiber.

**Table 2:** Phenols, Flavonoids, and Tannins content in seven invasive weeds from South-Central Montana.

	Species ^2^								
Secondary Metabolite ^1^	BURD	CAN	DOCK	KOC	LAMB	PIG	RUSS	SEM ^3^	*p*-Value
TPC, mg GAE/g	360.08 ^a^	28.70 ^e^	184.68 ^bc^	172.69 ^c^	171.14 ^c^	69.05 ^d^	45.79 ^de^	8.23	<0.001
TFC, mg QE/g	168.40 ^ab^	19.14 ^b^	261.12 ^a^	ND	78.75 ^b^	65.63 ^b^	24.71 ^b^	36.37	0.005
QTV, mg CE/g	180.45 ^c^	0.40 ^e^	274.39 ^a^	24.57 ^d^	222.32 ^b^	23.99 ^d^	179.91 ^c^	3.96	<0.001

^1^ TPC = Total Phenolic Content; TFC = Total Flavonoid Content; QTV = Quantified Tannin Value; CE = Catechin Equivalents. Percentages reflect dry matter (% DM); GAE = Gallic Acid Equivalents; QE = Quercetin Equivalents; ^2^ CAN = Canada Thistle. BURD = Common Burdock. KOC = Kochia. DOCK = Curly Dock. PIG = Prostate pigweed. LAMB = Lambsquarter. RUSS = Russian thistle. ^3^ SEM = standard error of the mean (*n* = 3 plants/species). Means with different letters in the same row were significantly different according to Tukey’s test (*p* < 0.05). ND = Not detected with the TFC method.

**Table 3:** Volatile Fatty Acid concentration and molar proportion after the addition of 20% of incubated DM of seven invasive weeds from South-Central Montana.

Item ^1^	Species ^2^									
*mM*	CON	CAN	BURD	DOCK	KOC	LAMB	RUSS	PIG	SEM ^3^	*p*-Value
Acetate	40.1 ^a^	39.02 ^a^	39.96 ^a^	37.16 ^a^	28.07 ^b^	38.24 ^a^	38.87 ^a^	36.06 ^a^	1.605	0.021
Propionate	16.46 ^bc^	17.56 ^ab^	18.46 ^a^	17.71 ^ab^	16.75 ^b^	15.66 ^c^	16.77 ^bc^	13.69 ^d^	0.445	<0.01
Butyrate	6.5 ^b^	6.38 ^bc^	6.28 ^bc^	6.95 ^a^	6.34 ^b^	5.97 ^bc^	6.09 ^b^	5.92 ^c^	0.195	0.049
BCVFA	2.71 ^a^	2.66 ^a^	2.69 ^a^	2.38 ^b^	2.72 ^a^	2.5 ^ab^	2.41 ^b^	2.18 ^b^	0.056	0.011
Tot	65.76 ^a^	65.63 ^a^	67.39 ^a^	64.2 ^a^	53.88 ^b^	62.37 ^a^	64.14 ^a^	57.85 ^ab^	1.874	0.041
A:P	2.4 ^a^	2.2 ^ab^	2.2 ^b^	2.1 ^b^	1.7 ^c^	2.4 ^a^	2.3 ^ab^	2.6 ^a^	0.102	0.039
***mol/100 mol***										
Acetate	60.97 ^a^	59.46 ^a^	59.3 ^a^	57.89 ^a^	52.1 ^b^	61.32 ^a^	60.61 ^a^	62.34 ^a^	3.270	0.069
Propionate	25.03 ^ab^	26.76 ^ab^	27.39 ^ab^	27.58 ^ab^	31.08 ^a^	25.1 ^ab^	26.14 ^ab^	23.66 ^b^	1.674	0.049
Butyrate	9.88 ^b^	9.72 ^b^	9.32 ^b^	10.82 ^ab^	11.77 ^a^	9.58 ^b^	9.49 ^b^	10.24 ^b^	0.645	0.041
BCVFA	4.12 ^b^	4.06 ^b^	3.99 ^b^	3.71 ^b^	5.04 ^a^	4.01 ^b^	3.76 ^b^	3.77 ^b^	0.243	0.048

^1^ BCVFA = Branched Chain VFA (Isobutyrate, Isovalerate, 2-Methyl-butyrate). A:P = Acetate-to-propionate ratio. ^2^ CAN = Canada Thistle. BURD = Common Burdock. KOC = Kochia. DOCK = Curly Dock. PIG = Prostate pigweed. LAMB = Lambsquarter. RUSS = Russian thistle. ^3^ SEM = standard error of the mean (*n* = 5/species). Means with different letters in the same row were significantly different according to Tukey’s test (*p* < 0.05).

**Table 4:** Ruminal fermentation parameters after the addition of 20% incubated DM of seven invasive weeds from South-Central Montana.

	Species ^2^									
Item ^1^	CON	CAN	BURD	DOCK	KOC	LAMB	RUSS	PIG	SEM ^3^	*p*-Value
Gas pressure, PSI	13.91 ^ab^	13.36 ^b^	13.61 ^b^	11.96 ^c^	14.12 ^a^	13.52 ^b^	13.44 ^b^	13.78 ^ab^	0.542	0.001
Total Gas, mmol	5.39 ^b^	5.00 ^b^	5.32 ^ab^	4.97 ^b^	5.43 ^a^	5.30 ^ab^	5.28 ^ab^	5.36 ^ab^	0.181	<0.01
CH_4_, mL	0.87 ^ab^	0.83 ^ab^	0.81 ^b^	0.72 ^c^	0.90 ^a^	0.81 ^b^	0.80 ^b^	0.854 ^ab^	0.043	<0.001
CH_4_, mL/g IDM	1.24	1.19	1.16	1.03	1.29	1.16	1.14	1.22	0.022	0.398
CH_4_, mL/g FOM	3.38 ^a^	2.96 ^b^	2.63 ^c^	2.73 ^c^	3.43 ^a^	3.08 ^b^	2.72 ^c^	3.04 ^b^	0.084	<0.01
pH	6.71	6.83	6.90	6.89	6.86	6.89	6.90	6.812	0.024	0.781
NH_3_-N, mM	6.96 ^a^	6.37 ^ab^	6.81 ^a^	4.67 ^c^	5.56 ^bc^	6.39 ^ab^	5.84 ^b^	5.78 ^b^	0.492	0.035
IVOMD, %	41.31	44.69	48.96	41.61	42.03	42.46	47.45	44.65	2.590	0.230

^1^ CH_4_ = Methane; IDM = incubated DM; FOM = Fermented Organic Matter; NH_3_-N = Ammonia nitrogen; IVOMD = In vitro Organic Matter digestibility ^2^ CON = Control treatment containing hay. CAN = Canada Thistle. BURD = Common Burdock. KOC = Kochia. DOCK = Curly Dock. PIG = Prostate pigweed. LAMB = Lambsquarter. RUSS = Russian thistle. ^3^ SEM = standard error of the mean (*n* = 5/species). Means with different letters in the same row were significantly different according to Tukey’s test (*p* < 0.05).

**Table 5:** Phenols, Flavonoids and Tannins content in the extracts from the selected invasive species.

	Species ^2^							
	BURD		DOCK					
	Portion		Portion			*p*-Value ^4^		
Item ^1^	Leaf	Infl	Leaf	Infl	SEM ^3^	Spec	Port	S × P
TPC, mg GAE/g	158.83 ^a^	96.17 ^b^	47.88 ^c^	56.18 ^c^	5.01	<0.01	<0.01	<0.01
TFC, mg QE/g	95.87	47.03	64.82	140.55	24.96	0.322	0.667	0.056
QTV, mg CE/g	63.32 ^c^	44.53 ^d^	77.51 ^b^	157.08 ^a^	2.62	<0.01	<0.01	<0.01
Yield (mg/g)	107.31	98.12	81.47	111.32	9.72	0.523	0.301	0.058

^1^ TPC = Total Phenolic Content; TFC = Total Flavonoid Content; QTV = Quantified Tannin Value; CE = Catechin Equivalents. Percentages reflect dry matter (% DM); GAE = Gallic Acid Equivalents; QE = Quercetin Equivalents; Yield = solid extract recovered after Ethanolic extraction. ^2^ BURD = Common Burdock; DOCK = Curly Dock; Leaf = Ethanolic extract from leaves; Infl = Ethanolic extract from Inflorescence. ^3^ SEM = standard error of the mean (*n* = 3/species-Portion treatment combination). ^4^ Observed significance levels for main effects of Species, portion, and their interaction (S × P); Means with different lowercase letters in the same row indicate differences between individual means due to a Species × Portion interaction according to Tukey’s test (*p* < 0.05).

**Table 6:** Volatile Fatty Acid concentration and molar proportion after the addition at 20% of Leaves and Inflorescence extracts from Common Burdock (BURD) and Curly Dock (DOCK)—Experiment 2.

		Species ^2^											
		BURD		DOCK									
		Portion		Portion			*p*-Value ^4^			*p*-Value Contrast ^5^ CON vs.			
Item ^1^	CON	Leaf	Infl	Leaf	Infl	SEM ^3^	Species	Portion	S × P	BURD		DOCK	
*mM*										Infl	Leaf	Infl	Leaf
Ace	35.42	31.89	31.23	30.54	32.46	1.152	0.095	0.272	0.150	0.503	0.355	0.010	0.270
Pro	13.83 ^b^	19.45 ^a^	18.75 ^a^	17.78 ^a^	18.21 ^a^	0.731	0.092	0.051	0.008	<0.01	<0.01	0.052	<0.01
But	5.43	5.81	5.64	6.14	6.01	0.288	0.203	0.642	0.419	0.341	0.079	0.026	0.041
BCVFA	2.34 ^A^	2.28 ^A^	2.29 ^A^	2.06 ^B^	1.97 ^B^	0.067	0.020	0.256	0.173	0.846	0.121	0.019	0.026
Total VFA	57.01 ^b^	59.43 ^a^	57.91 ^ab^	56.52 ^b^	58.65 ^a^	0.936	0.005	0.009	0.001	<0.01	0.020	0.083	<0.01
A:P	2.57	1.64	1.67	1.72	1.78	0.810	0.200	0.633	0.148	0.496	0.350	0.496	0.266
***mol/100 mol***													
Ace	62.12 ^A^	53.66 ^C^	53.93 ^B^	54.04 ^C^	55.35 ^B^	1.499	0.614	<0.01	0.837	<0.01	<0.01	<0.01	<0.01
Pro	24.25 ^C^	32.73 ^A^	32.37 ^B^	31.46 ^A^	31.04 ^B^	1.194	0.742	<0.01	0.259	<0.01	<0.01	0.014	<0.01
But	9.52 ^B^	9.78 ^A^	9.74 ^B^	10.87 ^A^	10.25 ^B^	0.586	0.050	<0.01	0.091	0.958	0.397	0.013	0.280
BCVFA	4.11 ^A^	3.83 ^A^	3.96 ^B^	3.64 ^A^	3.36 ^B^	0.130	0.602	<0.01	0.521	0.169	0.018	0.196	<0.01

^1^ Ace = Acetate; Pro = propionate; But = butyrate; BCVFA = Branched Chain VFA (Isobutyrate, Isovalerate, 2-Methyl-butyrate); Tot = Total VFA = Total volatile fatty acids; A:P = Acetate-to-propionate ratio. ^2^ BURD = Common Burdock; DOCK = Curly Dock; CON = Hay; Leaf = Ethanolic extract from leaves; Infl = Ethanolic extract from Inflorescence. ^3^ SEM = standard error of the mean (*n* = 3/species-Portion treatment combination). ^4^ Observed significance levels for main effects of Species, portion, and their interaction (S × P); Means with different lowercase letters in the same row indicate differences between individual means due to a Species × Portion interaction according to Tukey’s test (*p* < 0.05). Means with different uppercase letters in the same row indicate differences in the main effects (Species or Portion) after Dunnett’s adjustment. ^5^ Observed significance levels for pre-planned contrasts between the control diet (CON) and each Species × Portion combination.

**Table 7:** Ruminal fermentation parameters after the addition at 20% of Leaves and Inflorescence extracts from Common Burdock (BURD) and Curly Dock (DOCK)—Experiment 2.

		Species ^2^											
		BURD		DOCK						*p*-Value Contrast ^5^ CON vs.			
		Portion		Portion			*p*-Value ^4^			BURD		DOCK	
Item ^1^	CON	Leaf	Infl	Leaf	Infl	SEM ^3^	Spec	Port	S × P	Infl	Leaf	Infl	Leaf
Gas pressure, psi	13.68 ^a^	10.96 ^b^	11.12 ^b^	8.28 ^c^	8.16 ^c^	0.428	<0.01	0.654	0.48	<0.01	<0.01	<0.01	<0.01
Total gas, mmol	5.10 ^a^	4.60 ^b^	4.60 ^b^	4.10 ^c^	4.00 ^c^	0.090	<0.01	0.584	0.491	<0.01	<0.01	<0.01	<0.01
CH_4_, mL	0.86 ^a^	0.73 ^b^	0.74 ^b^	0.65 ^c^	0.64 ^c^	0.024	<0.01	0.601	0.395	<0.01	<0.01	<0.01	<0.01
CH_4_, mL/g IDM	1.22 ^A^	1.04 ^B^	1.05 ^B^	0.92 ^C^	0.91 ^C^	0.036	<0.01	0.414	0.397	<0.01	<0.01	<0.01	<0.01
CH_4_, mL/g FOM	3.47 ^a^	2.58 ^c^	2.72 ^bc^	2.94 ^b^	2.83 ^b^	0.091	0.037	0.042	0.021	<0.01	<0.01	<0.01	<0.01
pH	6.57 ^C^	6.81 ^B^	6.85 ^A^	6.76 ^B^	6.91 ^A^	0.036	0.859	<0.01	0.078	<0.01	<0.01	<0.01	<0.01
NH_3_-N, mM	7.64 ^A^	7.09 ^A^	6.45 ^A^	4.60 ^B^	4.66 ^B^	1.008	0.046	0.779	0.733	0.270	0.604	<0.01	<0.01
IVOMD, %	39.74 ^A^	45.13 ^A^	43.35 ^A^	34.92 ^B^	35.71 ^B^	2.26	0.002	0.830	0.581	0.053	0.183	0.081	0.140

^1^ CH_4_ = Methane; IDM = incubated DM; FOM = Fermented Organic Matter; NH_3_-N = Ammonia nitrogen; IVOMD = In vitro Organic Matter digestibility ^2^ BURD = Common Burdock. DOCK = Curly Dock. ^3^ SEM = standard error of the mean (*n* = 5/species). ^4^ Observed significance levels for main effects of Species, Portion, and their interaction (S × P); Means with different lowercase letters in the same row indicate differences between individual means due to a Species × Portion interaction according to Tukey’s test (*p* < 0.05). Means with different uppercase letters in the same row indicate differences in the main effects (Species or Portion) after Dunnett’s adjustment. ^5^ Observed significance levels for pre-planned contrasts between control diet (CON) and each Species × Portion combination.

## Data Availability

The data presented in this paper are available from the corresponding author on request.
